# Thinking Out-of-the-Box: A Non-Standard Application of Standard Pulse-Oximetry and Standard Near-Infrared Spectroscopy in a COVID-19 Patient

**DOI:** 10.1177/0885066620965167

**Published:** 2020-10-09

**Authors:** Patrick Schober, Erik J. Lust, Leo M. A. Heunks, Lothar A. Schwarte

**Affiliations:** 1 Department of Anesthesiology, Amsterdam University Medical Center, Vrije Universiteit Amsterdam, Amsterdam, The Netherlands; 2 Department of Intensive Care, Amsterdam University Medical Center, Vrije Universiteit Amsterdam, Amsterdam, The Netherlands

**Keywords:** near-infrared spectroscopy, NIRS, pulse oximetry, COVID, lingual, prone position

## Abstract

**Purpose:**

Purpose of this report is to describe the feasibility of lingual pulse oximetry and lingual near-infrared spectroscopy (NIRS) in a COVID-19 patient to assess lingual tissue viability after several days of mechanical ventilation in the prone position.

**Materials & Methods:**

In a COVID-19 ICU-patient, the tongue became grotesquely swollen, hardened and protruding from the oral cavity after 20 h of mechanical ventilation uninterrupted in the prone position. To assess the doubtful viability of the tongue, pulse-oximetric hemoglobin O_2_-saturation (SpO_2_; Nellcor, OxiMax MAX-NI, Covidien, MA, USA) and NIRS-based, regional tissue O_2_-saturation measurements (rSO_2_; SenSmart, Nonin, MN, USA) were performed at the tongue.

**Results:**

At the tongue, regular pulse-oximetric waveforms with a pulse-oximetric hemoglobin O_2_-saturation (SpO_2_) of 88% were recorded, i.e. only slightly lower than the SpO_2_ reading at the extremities at that time (90%). Lingual NIRS-based rSO_2_ measurements yielded stable tissue rSO_2_-values of 76-78%, i.e. values expected also in other adequately perfused and oxygenated (muscle-) tissues.

**Conclusion:**

Despite the alarming, clinical finding of a grotesquely swollen, rubber-hard tongue and clinical concerns on the adequacy of the tongue perfusion and oxygenation, our measurements of both arterial pulsatility (SpO_2_) and NIRS-based tissue oxygenation (rSO_2_) suggested adequate perfusion and oxygenation of the tongue, rendering non-vitality of the tongue, e.g. by lingual venous thrombosis, unlikely. To our knowledge, this is the first clinical report of lingual rSO_2_ measurement.

## Background

COVID-19 is primarily affecting the respiratory system, but complications may develop throughout the body as part of the disease (e.g., systemic inflammatory response with thromboembolic complications^1,2^) or as a side-effect of the ICU-treatment modalities. In this case report we describe a novel diagnostic approach to a rare complication, which could be either caused by the disease (e.g., venous thrombosis) or the ICU-treatment (e.g., prolonged prone positioning).

## The Case

**Figure 1.**
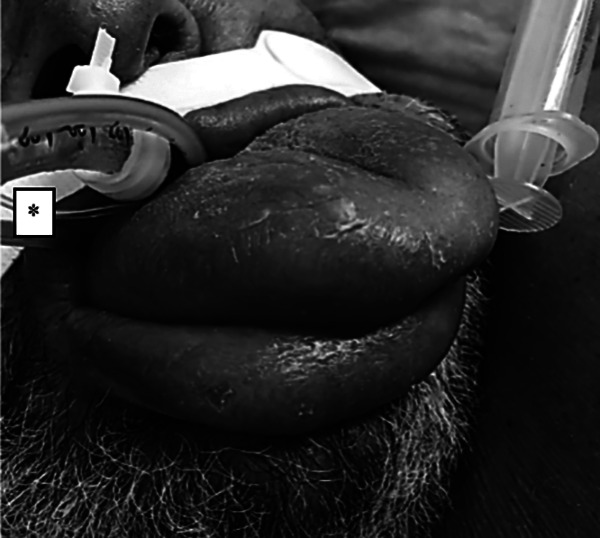
Covid-19 patient’s tongue protruding from the oral cavity after several days of mechanical ventilation with prone positioning. Photography taken hours after turning the patient back into supine position. To assess the questionable viability of the tongue, pulse-oximetric hemoglobin O_2_-saturation (SpO_2_) and regional tissue O_2_-saturation (rSO_2_, measured by near-infrared spectroscopy, NIRS) were successfully measured at the tongue. To our knowledge, this is the first clinical report of lingual rSO_2_ measurement. * Endotracheal tube with cuff-connector, gastric tube and esophageal pressure transducer.

In a COVID-19 ICU-patient (male, 71 years old, obesity with ∼110 kg, ex-smoker, no allergies), being ventilated uninterrupted in the prone position for 20 h, we encountered the following scenario:

The facial soft tissues, i.e. eye lids, periorbital soft tissue and lips were edematous to an extent judged compatible with the extended period of prone positioning. However, the patient’s tongue was swollen grotesquely, i.e. out of proportion of the other cranial soft tissues. The tongue was largely protruding from the mouth, even hours after test-wise placing the patient back in supine position (Figure 1). The massively enlarged tongue presented with a regularly colored surface, however, palpation revealed an inflexible and rubber-hard consistence.

The treating physicians were concerned about the viability of the tongue: Discussing that the swelling and hardening of the tongue may impair lingual perfusion and oxygenation (“compartment syndrome”), e.g. following lingual venous thrombosis, we thought to study arterial lingual perfusion and lingual tissue oxygenation.^3^

To study lingual arterial pulsatility, a disposable, adhesive, form-fitting pulse-oximetry-probe (Nellcor, OxiMax MAX-NI, Covidien, MA, USA) was wrapped around the edge of the tongue and affixed — a technique widely established in veterinary medicine. After a few seconds of auto-calibration, the monitor displayed a regular pulse-oximetry waveform with a pulse-oximetric hemoglobin-O_2_-saturation (SpO_2_) of 88%, being only slightly lower than the SpO_2_ reading at the extremities at that time (90%).

**Figure 2.**
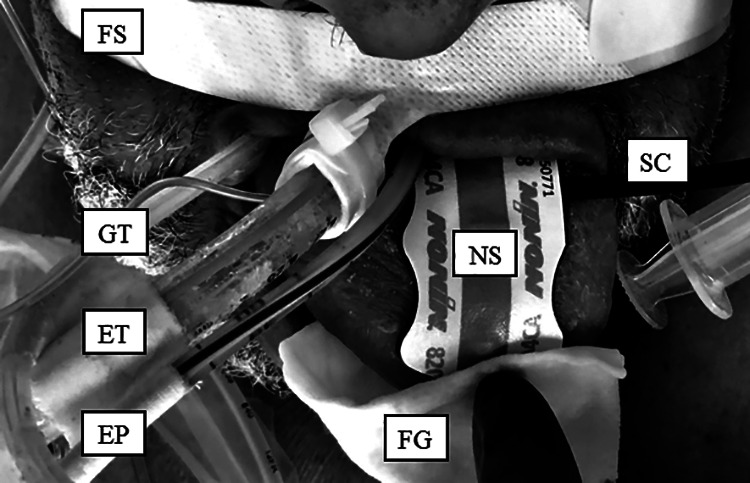
Covid-19 patient’s tongue protruding from the oral cavity after several days of mechanical ventilation with prone positioning. Photography taken hours after turning the patient back into supine position. To assess the doubtful viability of the tongue, regional tissue O_2_-saturation (rSO_2_, measured by near-infrared spectroscopy, NIRS) was successfully measured at the tongue. To our knowledge, this is the first clinical report of lingual rSO_2_ measurement. FS: Fixation-strap of the endotracheal tube; GT: Gastric tube; ET: Endotracheal tube; EP: Esophageal pressure transducer; FG: Fingertip and gauze, attaching the sensor to the tongue; NS: NIRS-sensor; SC: Sensor connector cable to the NIRS-monitor.

However, since presence of arterial pulsatility and arterial oxygenation (SpO_2_) alone do not guarantee adequate microvascular tissue oxygenation,^4^ e.g. in the theorized setting of lingual venous thrombosis, we intended to measure the regional hemoglobin-O_2_-saturation (rSO_2_) directly, i.e. by means of near-infrared spectroscopy (NIRS).^5^ Therefore, on the next day, with the patients tongue still being grotesquely enlarged, a clinical disposable NIRS probe (SenSmart, Equanox Advance, 8204CA, Nonin, MN, USA) was placed on the upper surface of the tongue (Figure 2). This probe-type has a 4 cm distance between light-source and light-detector for deeper tissue rSO_2_ measurements, compared to smaller pediatric probes. After brief equilibration, the connected NIRS monitor (SenSmart, Equanox X-100M, Nonin) displayed stable tissue rSO_2_-values of 76-78%, i.e. values that are expected in other adequately perfused and oxygenated (muscle-) tissues.^5^

Systemic hemodynamics at the time of lingual NIRS-measurement included an arterial blood pressure of ∼130/60 mmHg and a heart rate of ∼75 bpm (sinus rhythm). The patient was ventilated in “pressure control” mode, with a PEEP of 12 mbar and a FiO_2_ of 0.55, yielding an extremity SpO_2_ of ∼90%.

## Discussion & Conclusion

Despite the alarming, clinical finding of a grotesquely swollen, rubber-hard tongue, our measurements of both arterial pulsatility (SpO_2_) and NIRS-based tissue oxygenation (rSO_2_) suggested adequate perfusion and oxygenation of the tongue, rendering non-vitality of the tongue, e.g. by lingual venous thrombosis, unlikely.

**Figure 3.**
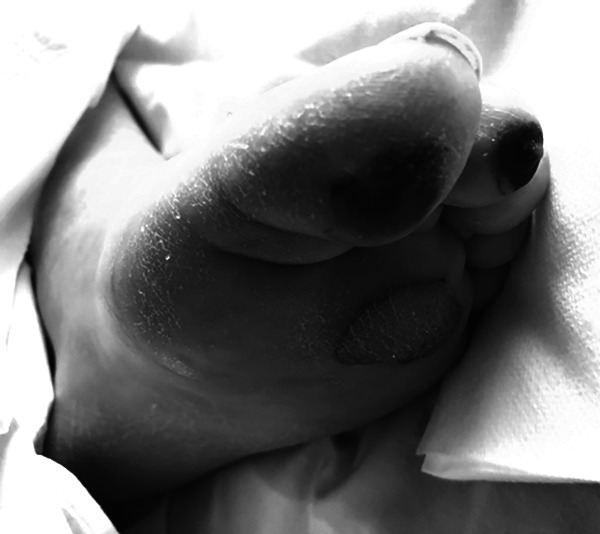
Plantar aspect (left foot) of the described Covid-19 ICU-patient. Several Covid-19 typical, avital spots, are visible. The etiology of these lesions is multifactorial, including endotheliitis and a thrombotic component. These factors were also initially discussed in the etiology of the hardened, swollen tongue of this patient, i.e. as a sequelae of endotheliitis, lingual thrombosis, and suspected tissue ischemia.

COVID-19 ICU-patients, such as the described patient, present several risk factors of impaired lingual viability: The COVID-19 disease itself triggers a general prothrombotic state, partly caused by endotheliitis and vasculitis, with (acro-) ischemic complications,^6,7^ also identified in the presented patient (Figure 3).

Recently, in COVID-19 also a Kawasaki-vasculitis related pathology has been proposed,^8^ and notably a Kawasaki-vasculitis derived complication is lingual ischemia.^9^ Furthermore, already various non-COVID-19 types of septic and cardiogenic shock have been associated with ischemia and even necrosis of the tongue.^10–12^

In addition, treatment modalities of COVID-19 patients jeopardize the tongue’s viability: Hypoperfusion of the tongue has been attributed to vasoactive drugs, such as vasopressin-analogues^13,14^ or catecholamines like noradrenaline, a vasopressor also used in the described COVID-19 patient.

Trans-orally positioned devices, e.g. tubes and lines, can compress the tongue and lingual vessels.^15–18^ Compressing lingual vessels may cause congestion, swelling and even necrosis of the tongue.^19^ In our case, an endotracheal tube, a gastric tube and an esophageal pressure-transducing tube (supporting advanced mechanical ventilation) were in place for several days, which may have contributed to the tongue’s swelling (Figures 1 and 2).

Finally, prolonged mechanical ventilation in the prone position may promote swelling of the tongue by gravitational hypostasis. Whether a single factor or combined factors^20^ caused the grotesque swelling of the tongue in our case, remains unclear.

Pulse oximetry is a standard technique to measure arterial hemoglobin oxygen saturation (SpO_2_), usually *via* a finger clip. These clips contain both a red LED (emitter) and an opposing light sensor (receiver). The arterial signal portion, required to calculate the arterial saturation, is computed from the pulsatile changes of total light absorption, i.e. slightly more light absorbing blood is present within the finger during arterial influx, thus more light is being absorbed by hemoglobin.^3^

In contrast to these pulsatile changes in total light absorption, the spectral changes of hemoglobin light absorption (i.e., the “color”) are used to determine the hemoglobin oxygen saturation: In commonly exploited wavelength ranges (e.g., 600-1000 nm), oxygenated hemoglobin shows less absorption at shorter wavelengths (up to ∼805 nm), but more absorption at longer wavelengths, compared to de-oxygenated hemoglobin. Measuring at wavelengths with those diverging optical properties of oxygenated and de-oxygenated hemoglobin allows the computation of hemoglobin oxygen saturation.^5^

**Figure 4.**
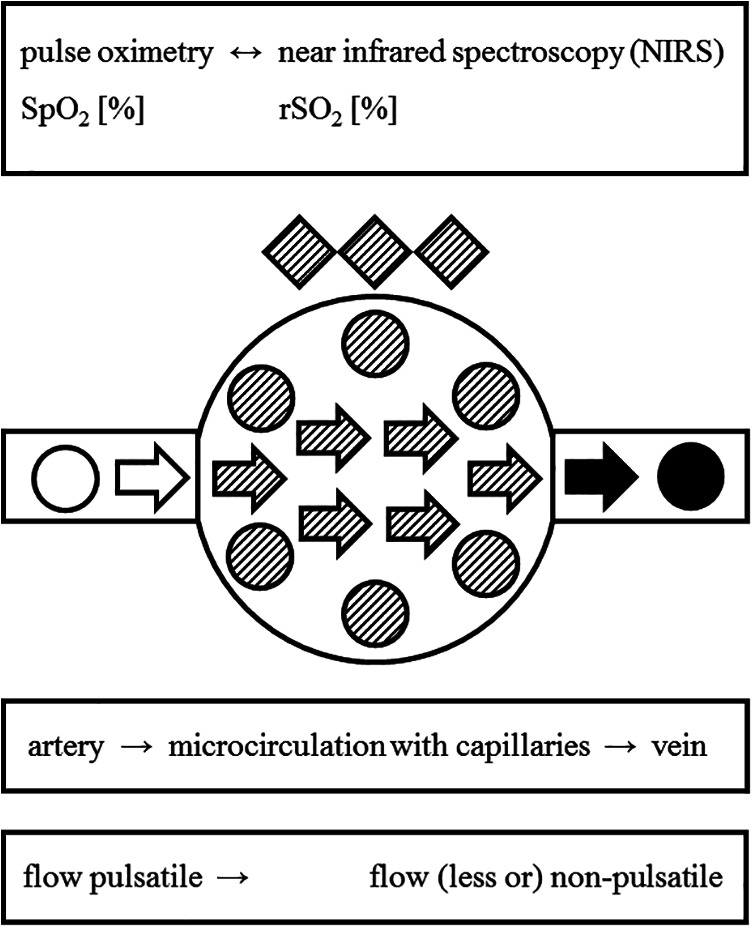
Differences between pulse oximetry and near infrared spectroscopy (NIRS). Within the functional compartments of the circulation, pulse oximetry assesses the pulsatile, predominantly arterial, hemoglobin oxygen saturation (SpO_2_, left side of figure and textboxes), typically using (near-) infrared LED-light of 660 and 940 nm. In contrast, NIRS assesses the oxygen saturation within the entire regional (micro-) circulation (rSO_2_), including capillaries and veins (mid and right portion of figure and textboxes). In muscle cells, NIRS also assesses the oxygen saturation of myoglobin (dashed rhombs), thus contributing to the rSO_2_ value. Depending on the specific NIRS-device, the applied wavelengths may differ, e.g. 730, 760, 810, and 880 nm for the applied NIRS-system. Arrows: direction of blood flow; circles: erythrocytes; rhombs: muscle cells; shading: state of oxygen saturation, i.e. high (empty symbols), medium (dashed symbols) and low oxygen saturation (filled symbols).

While pulse oximetry rather selectively measures arterial hemoglobin oxygen saturation, NIRS measures overall regional tissue oxygen saturation (rSO_2_), mainly comprising of microvascular hemoglobin and muscle myoglobin oxygen saturation (Figure 4).^5^ Current NIRS monitors discriminate light paths from different tissue depth, allowing for a rather selective tissue oxygenation measurement at deeper tissue layers, e.g. the cerebral cortex instead of the overlaying bone and skin layers.

Lingual pulse-oximetry is used in veterinary medicine,^21^ but very seldom reported in humans. With exception,^22^ the few (case-) reports published are restricted to the pediatric population.^23–25^ Moreover, while in those cases lingual pulse-oximetry is merely using the alternative location to assess systemic SpO_2_, our approach to assess arterial pulsatility and SpO_2_ to check the viability of the “organ tongue” has not been reported before.

To the best of our knowledge, the clinical application of lingual NIRS-based tissue oximetry has not been described before.

The application of pulse oximetry and NIRS to assess lingual tissue viability was prompted by the presented COVID-19 case, but these techniques can be used also to assess regional perfusion and oxygenation in other body regions. Pulse oximetry is used to monitor extremity perfusion in trauma patients, e.g. after suspected arterial injury. Also in elective vascular surgery patients (e.g., with intermittent claudication), pulse oximetry is applicable to monitor (re-)perfusion after arterial bypass grafting or arterial de-obstruction. Pulse oximetry in those settings is usually applied in conjunction with other diagnostic techniques.

The clinical use of lingual NIRS to assess tissue viability has, to our knowledge, not been described before. However, NIRS is used to assess tissue oxygenation of several other body regions.^26,27^ NIRS is advocated to monitor tissue oxygenation of the brain, specifically the frontal cortex, e.g. in cardiac surgery. In premature and critical ill neonates, NIRS is advocated for transcutaneous measurement of visceral oxygenation, e.g. of intestines and kidneys.

In general, this case demonstrates that out-of-the-box application of commercial standard equipment can help to solve non-standard clinical questions, exemplified in this COVID-19 patient.
